# Cross-Couplings via Electroreductive C–F, C–O, and C–S Bond Cleavage and Radical-Polar Crossover Mechanisms

**DOI:** 10.1021/acs.accounts.6c00486

**Published:** 2026-09-14

**Authors:** Luca Massaro, Helena Lundberg

**Affiliations:** Department of Chemistry, KTH Royal Institute of Technology, S-10044 Stockholm, Sweden

## Abstract

Electrochemical synthesis has emerged as a powerful platform for generating reactive intermediates under conditions that are difficult to achieve with conventional chemical reductants or oxidants. In particular, cathodic electron transfer to polarized C–X bonds can provide direct access to carbon-centered radicals, while a second electron transfer enables radical-polar crossover to carbanions that can be intercepted by suitable electrophiles. This umpolung sequence offers an attractive alternative to classical organometallic chemistry, allowing nucleophilic carbon intermediates to be generated without pyrophoric metals or strong bases. The apparent simplicity of this process, however, masks several selectivity challenges. Productive reactivity requires the substrate to be reduced and undergo mesolytic bond cleavage before the solvent, supporting electrolyte, electrophile, or product is consumed at the cathode. The resulting carbon-centered radical must thereafter accept a second electron faster than it undergoes hydrogen atom transfer, dimerization, or unproductive addition reactions. The resulting carbanion must subsequently be trapped within the same strongly reducing environment. Electrode materials, the anodic counter reaction, and concentration of the different species influence the kinetics and thermodynamics of electroreductive radical-polar crossover, requiring the matching of every component of the electrochemical setup rather than consideration of the substrate reduction potential alone. This Account describes our recent advances in addressing these challenges for electroreductive activation of C–F, C–O, and C–S bonds. In the context of C–F cleavage, trifluoromethyl arenes were converted into hydroxymethylated difluoromethyl arenes through selective monodefluorination followed by trapping with *N,N*-dimethylformamide. Mechanistic and electroanalytical studies revealed the privileged role of DMF and rationalized the high selectivity against over-reduction. In the context of C–O bond cleavage, deoxygenative strategies from alcohols and alcohol derivatives were explored. Under operationally simple galvanostatic conditions, benzylic and propargylic alcohols underwent hydrodeoxygenation and, in some cases, C–C bond formation with CO_2_ without prior derivatization. The reaction design was extended to deoxygenative C–Si bond formation in which hydrosilanes serve a dual function as activating agents and electrophilic reaction partners. Finally, aryl alkyl thioethers were developed as tunable alkyl radical precursors with the S-aryl group acting as an electroauxiliary that modulates the reduction potential and selectivity of bond cleavage. Depending on the reaction conditions and trapping partner, the resulting radical or carbanion intermediates provide access to C–H, C–D, C–C, and C–B bonds. For several of the protocols, borohydride oxidation was employed as an anodic counter reaction with a similar performance as the use of sacrificial metal anodes. Together, the highlighted studies show how electroreductive sequences with radical-polar crossover events can convert strong C–F, C–O, and C–S bonds into programmable synthetic handles. Future advances in mediators, electrocatalysts, and electrode design should further expand scope, improve chemoselectivity, and enable the construction of molecular complexity from abundant functional groups.

## Graphical abstract

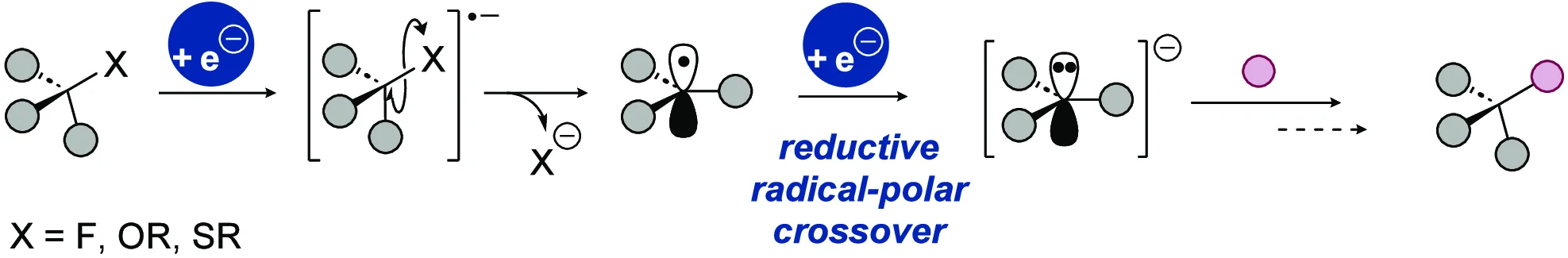

## Key References

-

Villo, P. ; Lill, M. ; Alsaman, Z. ; Soto Kronberg, A. ; Chu, V. ; Ahumada, G. ; Agarwala, H. ; Ahlquist, M. ; Lundberg, H. Electroreductive Deoxygenative C–H and C–C Bond Formation from Non-Derivatized Alcohols Fueled by Borohydride Oxidation. ChemElectroChem 2023, e202300420.^1^ Benzylic alcohols underwent electroreductive C–O bond cleavage to form alkanes or carboxylic acids via protonation of intermediate carbanions or their coupling with CO_2_, respectively.
-

Villo, P. ; Lill, M. ; Fan, Z. ; Breitwieser, K. ; White, J. , Pérez Morente, S. ; Ahlquist, M. ; Lundberg, H. Electrochemical Deoxygenative Silylation of Alcohols. Angew. Chem. Int. Ed. 2025, 64, e202508697.10.1002/anie.202508697PMC1245542740790906 ^2^ Benzylic and allylic alcohols underwent electrochemically driven deoxygenative cross-coupling with diphenylsilane, and reactivity trends were mapped.
-

Kuzmin, J. ; Röckl, J. L. ; Schwarz, N. ; Djossou, J. ; Ahumada, G. ; Ahlquist, M. ; Lundberg, H. Electroreductive Desulfurative Transformations with Thioethers as Alkyl Radical Precursors. Angew. Chem. Int. Ed. 2023, 62, e202304272.10.1002/anie.20230427237342889 ^3^ Aliphatic and benzylic aryl thioethers were electrochemically transformed into alkanes, deuterated alkanes, carboxylic acids, and Giese-type adducts, and structure-selectivity trends were explored for the S-aryl substituent.
-

Kuzmin, J. ; Margarita, C. ; Winter, J. ; Lundberg, H. Electrochemical Desulfurative Borylation of Thiols, Disulfides, Thioethers and Thioacetals. Nat. Commun. 2026, 17, 632.10.1038/s41467-025-67363-7PMC1281663641540045 ^4^ Thiols and their derivatives underwent desulfurative borylation with pinacolborane via an electroreductive route.
-

Shatskiy, A. ; Holczer, M. ; Winter, J. ; Fan, Z. ; Breitwieser, K. ; Lundberg, H. Selective Electrochemical Defluorinative Hydroxymethylation toward Difluoro-Substituted Alcohol Building Blocks. Org. Lett. 2026, 28, 7097–7103.10.1021/acs.orglett.6c01339PMC1327063342228768 ^5^ Trifluoromethylarenes underwent selective monodefluorination and cross-coupling with DMF to furnish hydroxymethylated difluoromethylarene products via a net-reductive, electrochemical route.

## 1 Introduction

Electrochemical generation of reactive species has become an important synthetic strategy for bond cleavage and bond formation. The appeal of the strategy relies on the new reactivity and selectivity that can be achieved, as well as sustainability aspects, economy and scalability. These features have spurred great interest from both academia and industry,^6–9^ the latter including BASF’s electrooxidative conversion of *p*-methoxytoluene to the dimethyl acetal of anisaldehyde and Monsanto’s electroreductive dimerization of acrylonitrile to adiponitrile.^10^ As for any redox reaction, an electrolytic transformation entails an oxidation and a reduction to occur simultaneously to keep the net number of electrons constant. Electrosynthetic redox reactions take place at the surface of the working electrodes: oxidations at the anode and reductions at the cathode. A wide variety of electrode materials are available and the choice can greatly affect the reaction outcome.^11^ If the electrosynthetic transformation is net oxidative, a sacrificial reductive transformation, such as hydrogen evolution, takes place at the cathode. For a net reductive transformation, a sacrificial oxidative transformation needs to occur at the anode and the use of an easily oxidized metal anode or organic additive is common for this purpose.^12–14^ A net oxidative and a net reductive process can also be coupled in paired electrolyses for enhanced energy and resource efficiency. For in depth discussions of the principles for organic electrosynthesis and tutorials on how to choose parameters, the reader is referred to other literature.^15–19^

Synthetic electrochemical transformations rely on the generation of reactive species at an electric potential related to the redox potential of the starting material in question.^20,21^ For direct electrolyses of organic molecules, the electron transfers typically proceed via one electron at a time to produce radical ion intermediates. These open shell species are commonly highly reactive and set off a chain of events that ultimately lead to the formation of the target product. In the context of reductive transformations, cathodically induced cleavage of polarized sigma bonds to furnish carbon-centered radicals is a strategy of great synthetic utility. The approach is particularly prominent for the cleavage of carbon-halide bonds for, e.g., selective dehalogenation of complex organic molecules,^22^ pollutant remediation^23–25^ as well as cross-electrophile couplings with CO_2_ or alkyl halides.^26–36^ In the classic scenario, reduction of the alkyl halide can proceed either through concerted dissociative electron transfer, in which electron transfer and bond cleavage occur in a single step, or through a stepwise sequence in which a discrete radical anion is formed and subsequently undergoes mesolytic bond scission to furnish a halide ion and a carbon-centered radical (Figure 1). The latter can thereafter react directly with another radical, with a SOMOphile such as an alkene, with a metal catalyst for subsequent cross-coupling,^37,38^ or undergo a second reductive electron transfer and furnish the corresponding carbanion upon a radical-polar crossover for subsequent reaction with an electrophile. In contrast to traditional methods for generating carbanions, such electrochemical generation via reductive radical-polar crossover avoids the hazardous use of pyrophoric metals like Li and Mg, strong bases such as alkyl lithium reagents or LDA, a feature that can enable new selectivities and enhanced functional group tolerance.^39^ While possible for open shell species situated in fully aliphatic positions,^33^ the radical-polar crossover route is favored for positions with adjacent groups that can stabilize the charged intermediate, such as π-systems or electron-withdrawing groups.^30,34^ The reactivity trends and operating principles for electroreductive bond cleavage and radical-polar crossover are analogous to those using chemical single electron reductants or photoredox catalysis. However, whereas stoichiometric chemical reductants and photocatalysts operate within potential windows defined by their intrinsic redox properties, electrochemistry provides a more modular platform in which the applied potential can be tuned to access increasingly reducing regimes. This can enable electron-transfer events to occur that are difficult to achieve using conventional reductants or photochemical mediators, while modulation of parameters such as current, electrode material, and reaction setup can provide additional control over rates and selectivities.^40^ In this Account, we highlight electroreductive strategies for the less explored C–F, C–O and C–S bond cleavage with concomitant cross-couplings, proceeding via radical-polar crossover mechanisms with particular emphasis of recent work from our lab.

**Figure 1:**
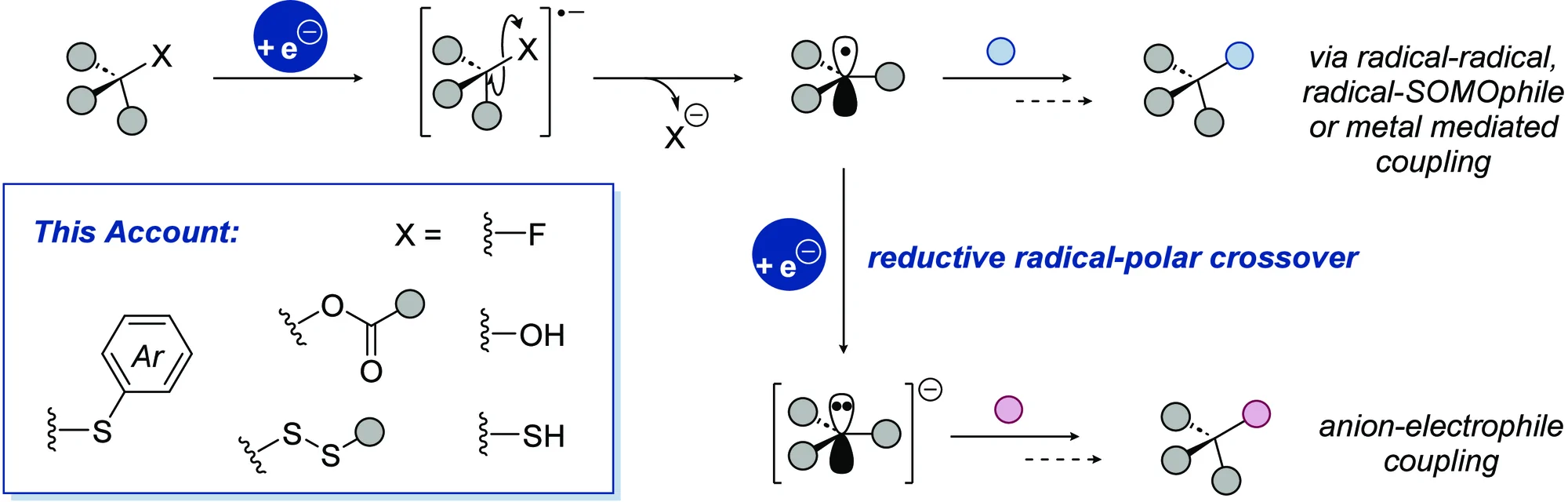
Reductive C–X bond cleavage and cross-coupling of reactive intermediates.

## 2 Carbanions via Reductive Cleavage of Polarized Sigma Bonds

### 2.1 C–F Bond Cleavage

In contrast to electroreductive cleavage of C–I, C–Br and C–Cl bonds, protocols for electroreductive cleavage of C–F bonds are less common. This lower abundance may be understood in light of their less reactive nature, reflected in highly negative reduction potentials and higher bond dissociation energies compared to their heavier congeners.^41,42^ Due to their relative inertness, reductive removal of fluorides is commonly not possible in absence of adjacent activating groups, such as carbonyl compounds or π-systems.^43–52^ For trifluoromethyl arenes, the C–F bond dissociation energy (BDE) and the rate of mesolytic bond cleavage in the corresponding radical anion decreases with the fluorination degree at a specific carbon,^53,54^ translating to easier removal of fluorides from an already partially defluorinated compound. In line with this trend, electroreductive protocols for full defluorination of trifluoromethyl arenes to the corresponding methyl arenes or coupled products are well-established.^55–58^ At the same time, the reduction potential of the fluorinated compound typically becomes more negative as the number of fluorides at the carbon center decreases.^54^ With control of the applied potential, either by potentiostatic electrolysis or close monitoring of the reaction progress under galvanostatic conditions, this feature can enable selective partial hydrodefluorination of trifluoromethyl arenes (Figure 2). Such partial hydrodefluorination is an important transformation class due to the increasing interest in difluoromethyl groups as bioisosteres for hydroxyl, thiol and methyl groups with relevance for pharmaceutical and agrochemical applications,^59,60^ and an elegant protocol was recently disclosed by Lennox and co-workers.^42^ In this case, high selectivity for difluoromethyl arene products was observed when 2 F charge was passed, whereas monofluoromethyl analogues could be obtained with further charge passed and increased concentration of additives.^42,61^ With a similar approach, partial hydrodefluorination of trifluoromethyl arenes to the difluorinated analogues was shown by Cheng and co-workers.^62,63^ Typically, acetonitrile, ammonia, D_2_O or the tetrabutylammonium ion of the supporting electrolyte served as proton/deuteron source for the defluorinated products.^55,57,62,63^

**Figure 2:**
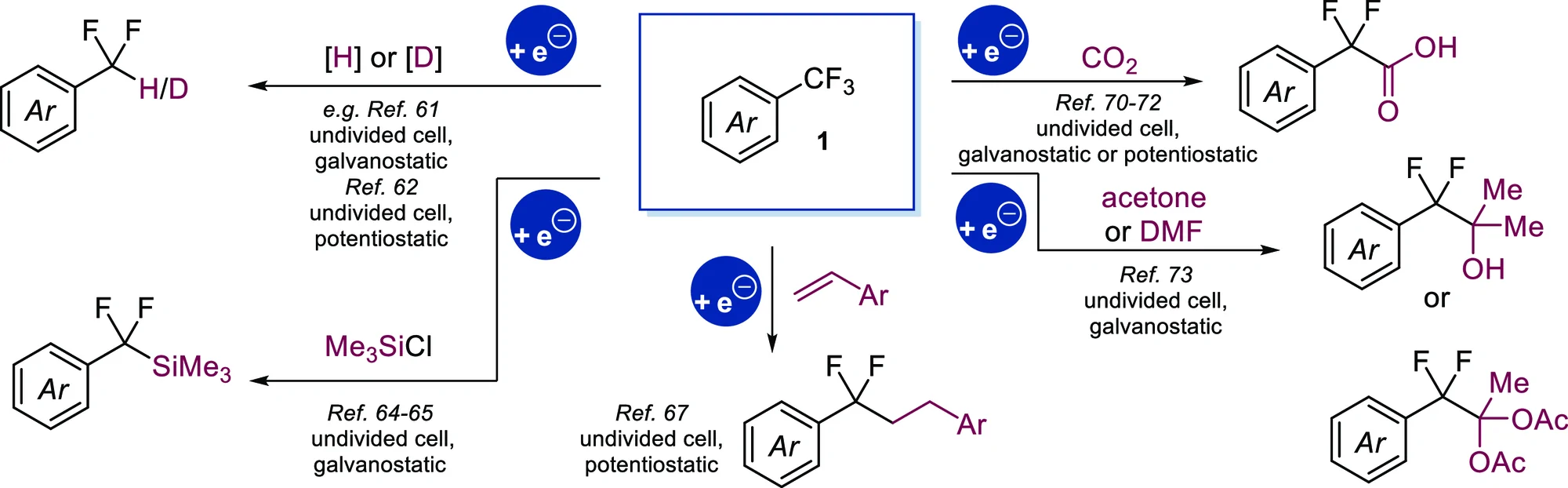
Electroreductive partially defluorinative cross-couplings.

Compared with selective hydrodefluorination, partial defluorination of trifluoromethyl arenes (**1**) with concomitant coupling to other species than protons/deuterons is considerably less explored. This limited prevalence may be understood in terms of the commonly poor resistance of electrophiles to the highly cathodic potentials required to set off the key C–F bond cleavage. Nevertheless, a few electrophile classes have been demonstrated to survive such reducing conditions. In addition to their use as fluoride trapping agents,^61^ silyl chlorides have been demonstrated to function as robust electrophiles in defluorinative sequences. For example, monodefluorinative silylation was demonstrated by Clavel, Bordeau and co-workers from trifluoromethyl arenes (Figure 2),^64,65^ with similar reactivity demonstrated by Uneyama for defluorinative silylation of trifluoromethylated carbonyl compounds.^66^ In addition, alkenes was recently disclosed by Cheng and co-workers to serve a cross-coupling partners for monodefluorinative C–C bond formation from trifluoromethylarenes (Figure 2).^67^ With a fully defluorinative outcome, Lyu and co-workers disclosed a Matteson-type borylation/homologation protocol that proceeds via radical-polar crossover and coupling with alkyl borate esters.^68^ Finally, certain classes of carbonyl compounds have been demonstrated feasible as coupling partners. In a fully defluorinative sequence, Zhou, Lei and Yang recently demonstrated that trifluoro- and difluoromethyl arenes could be efficiently defluorinated and coupled with CO_2_ to furnish 1,1-deuterocarboxylated products, using D_2_O as proton source.^69^ Partial defluorination with concomitant C–C bond coupling using CO_2_ as electrophile has also been reported, including contributions from the groups of Senboku, Guirado and Meanwell (Figure 2).^70–72^ A few examples of partial defluorocarboxylation of trifluoromethylarenes were also demonstrated by Troupel and co-workers, in addition to a limited number of examples of the formation of difluorinated tertiary alcohols and acetals from acetone and *N,N*-dimethylformamide (DMF) (Figure 2).^73^

We recently demonstrated that DMF can serve as hydroxymethylating agent,^5^ selectively furnishing difluoromethyl arene products with the core functional unit found in biologically active compounds (Figure 3).^74,75^ Tetrabutylammonium borohydride (TBABH_4_) was employed as a soluble sacrificial reductant, with anodic borohydride oxidation serving as the counter reaction for this net-reductive transformation (*vide infra*).^12,76,77^ The trifluoromethyl arene substrates (**1**) undergo two consecutive electron transfers to furnish the key carbanionic intermediate. This species attacks DMF and releases a dimethylamide ion and an aldehyde intermediate that subsequently undergo further reduction to the corresponding hydroxymethylatated difluoromethylarene (Figure 3). In this case, DMF could be used as solvent or cosolvent in toluene or THF with similar results. Interestingly, other potential carbonyl coupling partners such as dimethyl carbonate and methyl formate resulted in considerably lower yields of the hydroxymethylated compound **2**, whereas *N*-methyl-2-pyrrolidone and 1,3-dimethyl-2-imidazolidinone failed to form coupling products. Similarly, the related *N,N*-dimethylacetamide (DMAc) failed to form coupling products. To probe the reason behind the latter, DFT calculations were carried out for the coupling of the key difluorinated carbanion and each acceptor. While the barrier for the C–C coupling was found to be of similar magnitude in both cases, only the coupling with DMF was found to be an exergonic process. For DMAc, deprotonation of the α-position was instead found to be the thermodynamically favored pathway, thereby rationalizing the observed privileged role of DMF as acceptor (Figure 3). The ability of DMF as cross-coupling partner is notable, provided that similar electroreductive protocols for trifluoromethyl arenes carried out in DMF have shown to selectively furnish hydrodefluorinated products,^62,63,78^ highlighting the importance of tuning of reaction conditions. In our undivided setup with 2.5 F applied charge and tetrabutylammonium borohydride (TBABH_4_, 0.1 M) as sacrificial reductant, carbon-based electrodes proved optimal and two sets of conditions were established: graphite electrodes and a current density of 6.9 mA/cm^2^ at room temperature (Conditions A) or a graphite cathode and a BDD anode with a current density of 32.9 mA/cm^2^ at 0 °C (Conditions B). Exchange of the C-based anode for Mg or Zn with removal of TBABH_4_ failed to furnish the target product **2a** or defluorinated alkane side-products. Using Conditions A or B, a selection of difluorohydroxymethyl arene products formed in moderate to good yields with the overdefluorinated products rarely observed. To rationalize this high selectivity, cyclic voltammetry (CV) measurements were carried out, demonstrating that the model substrate **1a** undergoes reductive electron transfer events prior to that of the corresponding hydroxymethylated product **2a**. As such, the higher cathodic stability of the product is likely the reason behind the excellent chemoselectivity observed for the transformation.

**Figure 3:**
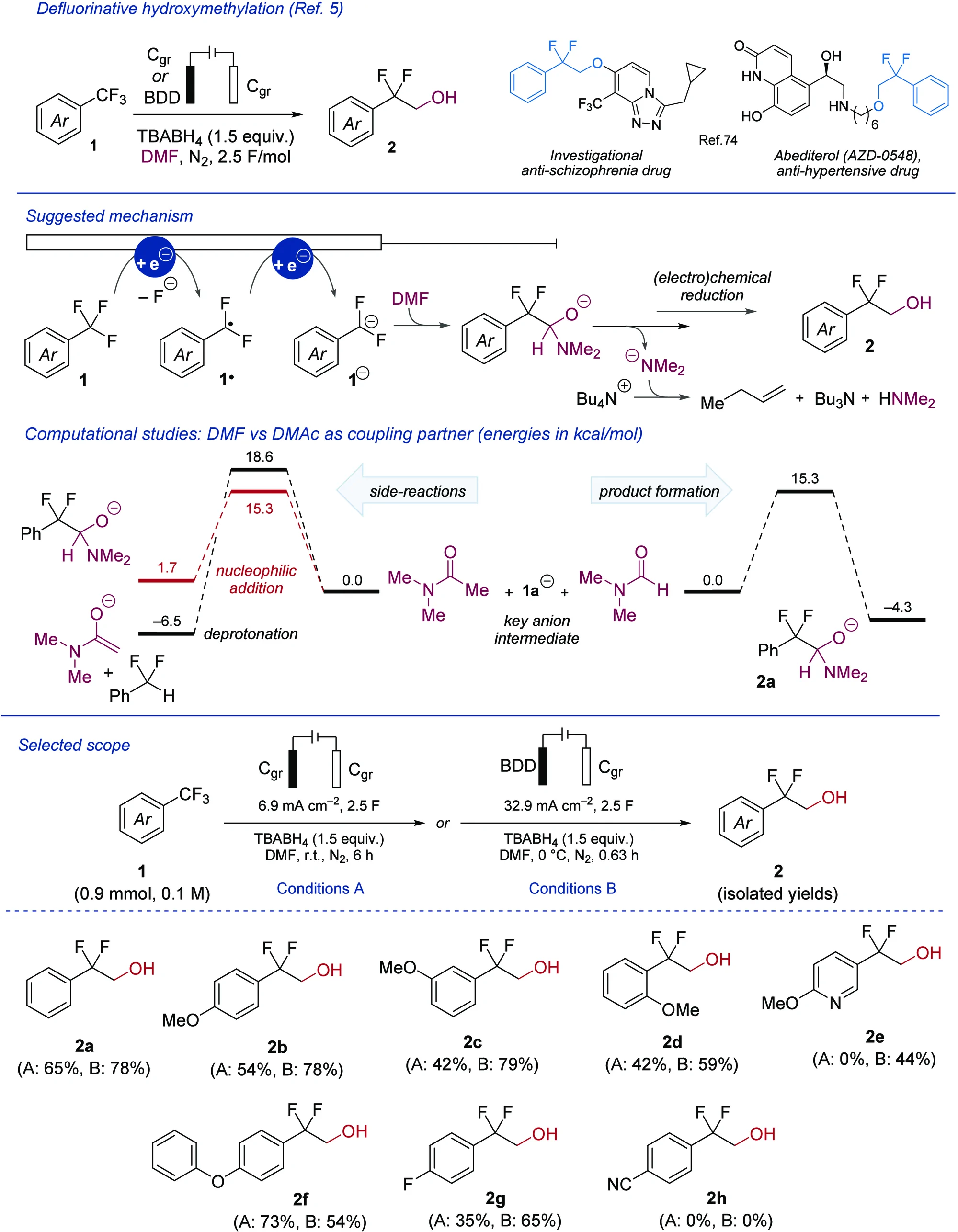
Defluorinative hydroxymethylation of trifluoromehylarenes. A. Reaction overview. B. Proposed electroreductive radical–polar crossover mechanism. C. Computed comparison of DMF and DMAc as coupling partners. D. Reaction conditions and selected scope. Adapted with permission from Ref. 5, licensed under CC BY 4.0. Copyright 2026 Americal Chemical Society/the authors.

### 2.2 C–O Bond Cleavage

Alcohols and their derivatives are highly abundant starting materials in organic compounds of both natural and synthetic origin. While typically weaker compared to the C–F bond, C–O bonds are associated with high BDEs and significant barriers for bond cleavage via both polar and radical routes.^53^ To overcome their synthetic inertness, alcohols are typically stoichiometrically functionalized to enable more facile C–O bond scission. In an electrochemical manifold, a variety of protocols have been developed over the years.^79–84^ With a few exceptions, including carbocationic routes and electrooxidative transformations via oxalate esters or phosphonium/sulfonium intermediates,^84–87^ these protocols typically proceed via net-reductive routes and have enabled C–O bond cleavage in, *e.g.,* ethers,^88,89^ sulfonate esters,^90–92^ and phosphinate, phosphate and phosphonate esters.^92–94^ Similarly, reductive C–O bond cleavage has been demonstrated by Markó and Lam from carboxylic esters.^95–98^ The Markó-Lam reaction typically furnishes alkanes, commonly believed to proceed from the intermediate radical via hydrogen atom transfer (HAT) (Figure 4A). However, π-activated alkyl radicals were proposed to undergo a second electron transfer to the corresponding carbanion able of subsequent attack onto carbonyl compounds.^97^ On a similar note, deoxygenative carboxylation of dibenzylacetates, carbonates and epoxides has been demonstrated via a similar radical-polar crossover route with attack of intermediate carbanions onto CO_2_ (Figure 4A).^99–102,88,89^ Recently, deoxygenative cross-electrophile coupling of benzylic ^*t*^Bu-benzoate esters and alkyl halides was reported by Wang, Zhang and co-workers.^103^ In this case, reductively induced C–O bond cleavage of Markó-Lam type furnished a radical intermediate that underwent further cathodic reduction to afford a stabilized carbanion, subsequently reacting with primary alkyl bromides via an S_N_2 pathway (Figure 4A). This radical-polar crossover mechanism is also presumed to operate for our recently disclosed deoxyborylation of benzyl esters **3** to furnish benzylpinacol borane **4a** (Figure 4B).^104^ In this case, only sufficiently electron-rich esters, displaying an irreversible redox behavior as assessed by cyclic voltammetry (CV), were found to undergo the targeted deoxygenative C–B bond formation. This finding was by computational means correlated to the stability of the esters’ corresponding radical anions. Specifically, it was found that esters that required a highly negative potential to convert to their charged open shell species displayed a higher degree of spin density on the carbonyl oxygen of the charged open shell species and a lower barrier for the C–O bond scission, compared to esters with a more anodic reduction potential. Furthermore, the key reductive radical-polar crossover of the benzyl radical was found to be less favored for the latter category of esters, a feature that was rationalized by the small difference in the reduction potential of the ester and that of the benzyl radical (Figure 4B). While yet to be expanded for esters with aliphatic alcoholic side chains, the study exemplifies the potential of Markó-Lam type reactivity for new classes of deoxygenative intermolecular transformations.

**Figure 4:**
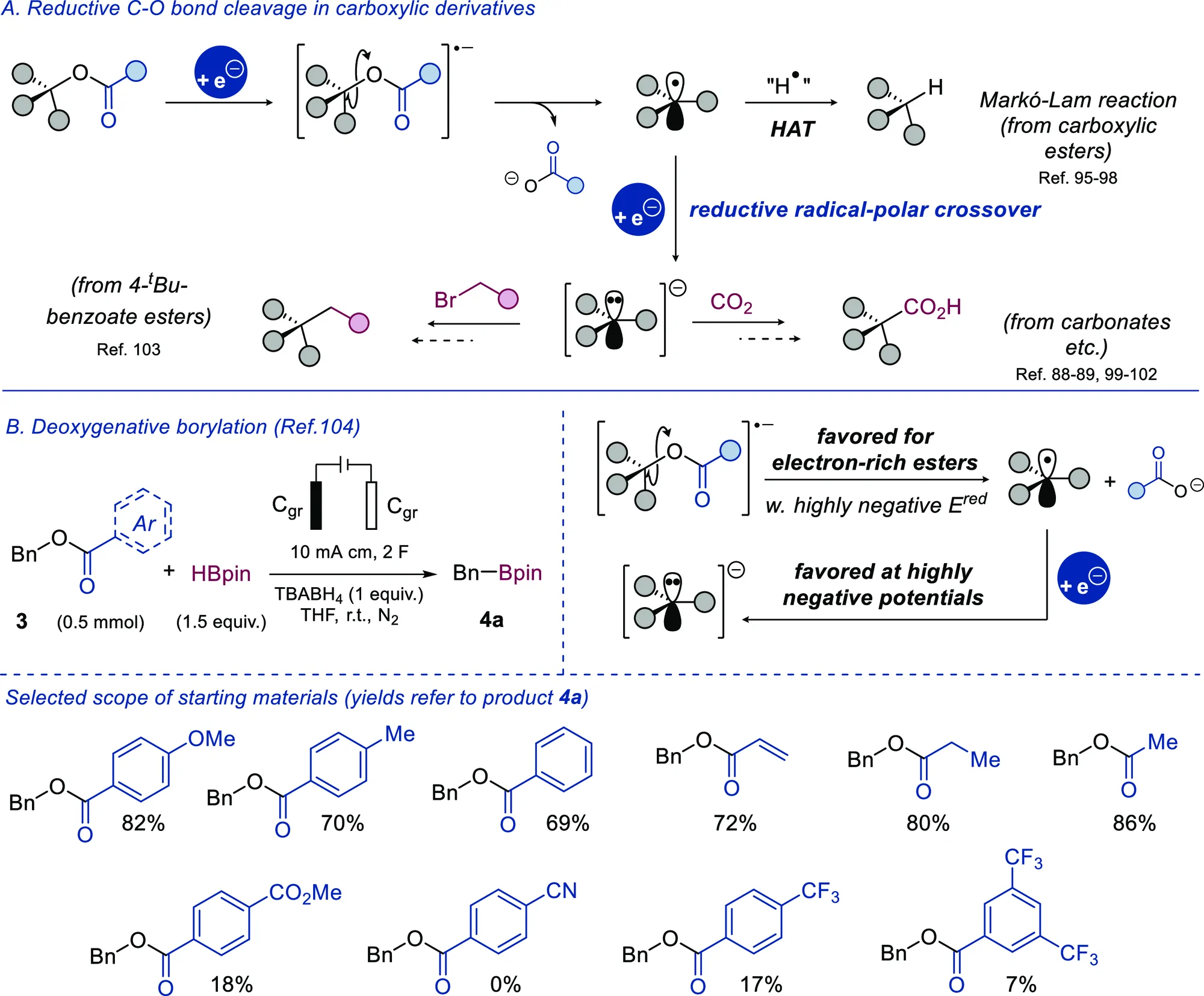
Deoxygenative transformations of carboxylic derivatives. A. C–O bond cleavage and coupling in carboxylic derivatives B. Deoxygenative borylation. Adapted with permission from Ref. 104 licensed under CC BY 4.0. Copyright 2026 American Chemical Society/the authors.

Due to their highly negative reduction potentials, electroreductive deoxygenation of native nonderivatized alcohols is challenging and remain limited to substrates with adjacent π-conjugated systems that shift the reduction potential to less negative values and stabilize high energy intermediates.^79–84,86^ Inspired by early works in the field,^106–113^ our lab developed a user-friendly protocol for hydrodeoxygenation of benzylic and propargylic alcohols (**5**) using graphite electrodes in a DMF/TBABH_4_ electrolyte under galvanostatic conditions.^1^ Under these conditions, primary, secondary and tertiary benzylic alcohols were hydrodeoxygenated to the corresponding alkanes (**6**) in good to excellent yields (Figure 5A), presumably following the mechanism previously suggested for Birch-type conditions.^114–116^ Furthermore, propargylic alcohols underwent deoxygenative reduction to the corresponding alkanes, as did cyclic and linear benzylic ethers. Recently, the latter was also demonstrated feasible in a recyclable deep eutectic electrolyte without the addition of supporting electrolyte.^117^ Deuterium-labeling experiments indicated that the quaternary tetrabutylammonium ion of the supporting electrolyte is likely to be the primary H-source, delivering a proton to the key carbanionic intermediate via Hofmann elimination.^118–120^ Switching the nitrogen atmosphere for CO_2_ enabled the formation of carboxylic acids (**7**) in up to moderate yields, with electron-poor benzylic substrates performing best. A limitation was observed for the highly electron-rich benzylic substrate that furnished hydrodeoxygenation product **6f** in 79% yield but did not provide the corresponding carboxylation product **7d**. This finding was rationalized as the result of the more anodic reduction potential of CO_2_ compared to the alcohol substrate, effectively affording preferential reduction of the former under the applied galvanostatic conditions.

**Figure 5:**
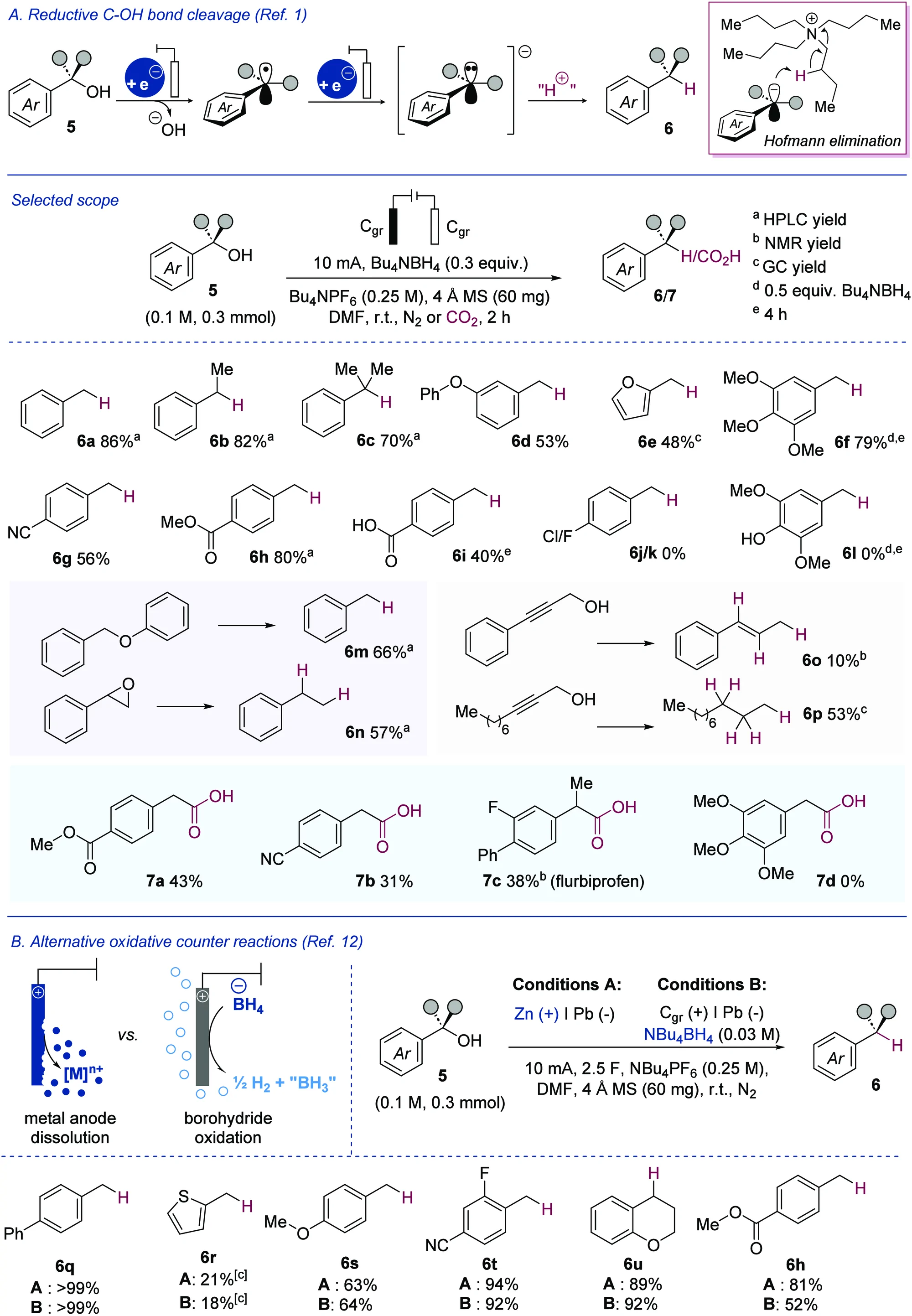
Deoxygenative cross-couplings of benzylic and propargylic alcohols. A. Hydrodeoxygenation and carboxylation of π-activated alcohols. B. Comparison of anode dissolution and borohydride oxidation as counter reactions. Adapted with permission from Ref. 1 and Ref. 12 licensed under CC BY 4.0. Copyright 2023/2025 Wiley/the authors.

In the screening for optimal reactions conditions for electroreductive hydrodeoxygenation, we realized that the efficiency of the anodic counter reaction was of great importance for the net reductive transformation, likely due to the facile oxidation of the alcohol substrate in lack of other anodic transformations taking place in the undivided cell. With inspiration from Huang and co-workers,^76,77^ TBABH_4_ was assessed as additive and proved to be highly useful for the overall transformation. A series of control reactions confirmed that hydride oxidation was likely the main counter reaction for the net cathodic transformation, effectively serving as the inverse of proton reduction. In a follow-up study, the generality of this borohydride oxidation strategy as anodic counter reaction was probed for a selection of net-cathodic transformations.^12^ For the alcohol reduction, similar yields of the alkane product were obtained using either the TBABH_4_ with a sacrificial Zn anode (Conditions A) or a graphite anode (Conditions B) (Figure 5B). This interchangeability was comparable to what was observed for hydrodesulfurization and desulfurative borylation of thioethers (*vide infra*). Only in the case of the ester-decorated compound **6h** was a decrease in yield observed for the borohydride system, possibly due to chemical side-reactions of the ester with the tentatively formed borane byproduct. Such a borane may also be able to react with the alcohol substrate and form borate esters, species that may be anticipated to undergo reduction at less cathodic potentials compared to the alcohol itself. To probe whether such borate esters were likely intermediates in the hydrodeoxygenation, a series of control reactions were conducted. While *ex situ* formed borate ester of *p*-phenylbenzyl alcohol was indeed possible to convert to the corresponding alkane, the forcing conditions required for its synthesis and the lack of data to support its presence in reaction mixtures could not strengthen this mechanistic pathway.

While borate esters could not be confirmed as intermediates in our hydrodeoxygenation protocol, such species were identified as key species by Lin and co-workers for their deoxygenative borylation of benzylic and allylic alcohols and derivatives with pinacolborane (HBpin), proceeding via a electroreductive radical-polar crossover route (Figure 6A).^121,122^ Similarly, an *in situ* formed intermediate of the alcohol and the electrophilic cross-coupling partner was proposed for the electroreductive deoxygenative carboxylation of benzylic alcohols with CO_2_, reported by Senboku and co-workers.^123,124^ Recently, the same was proposed for deoxygenative C–Si coupling of π-activated alcohols with hydro- and chlorosilanes by us and the groups of Xu and Lin (Figure 6A).^2,125,126^ In our case, SiH_2_Ph_2_ was used as coupling partner for benzylic and allylic alcohols, furnishing deoxygenated silane products in good to excellent yields using an undivided cell and galvanostatic electrolysis in a THF/TBAPF_6_ electrolyte (Figure 6B). In agreement with the studies of Xu and Lin,^125,126^ control reactions suggested that the silane reacts chemically with the alcohol to furnish a silyl ether that subsequently undergoes a reductive electron transfer to furnish a carbon-centered radical. A second reductive electron transfer sets off the radical-polar crossover and furnishes the carbanion that reacts with the Si-electrophile (Figure 6A). Interestingly, the trend with respect to yield of silylated product **8** was opposite to that of carboxylation with CO_2_: while the latter required electron-poor substrates to furnish decent yields of the coupled product **7**, the former required electron-rich substrates. To probe the origin of this difference, we carried out DFT calculations to assess the reaction energies and barriers for the key anionic cross-coupling step. Interestingly, it was found that the barriers and reaction energies were lower for electron-rich substrates than for electron-poor analogues for both C–C and C–Si coupling (Figure 6C), thereby failing to explain the differences in observed trends in yield. Instead, the observed difference was concluded to be the result of relative reduction potentials of starting materials and coupling partners. In the case of silylation, the silyl ether intermediate for the electron-rich benchmark substrate *p*-methoxybenzyl alcohol undergoes a cathodic event prior to the silane coupling partner, whereas only electron-poor alcohols would undergo reductive electron transfer at a more anodic potential compared to CO_2_. As such, selective cross-coupling relies on matched reduction potentials of the substrate and the electrophilic coupling partner to ensure that the latter is more cathodically stable than the former (Figure 6C), whereas the opposite scenario will lead to preferential reduction of the coupling partner and subsequent side-product formation.

**Figure 6:**
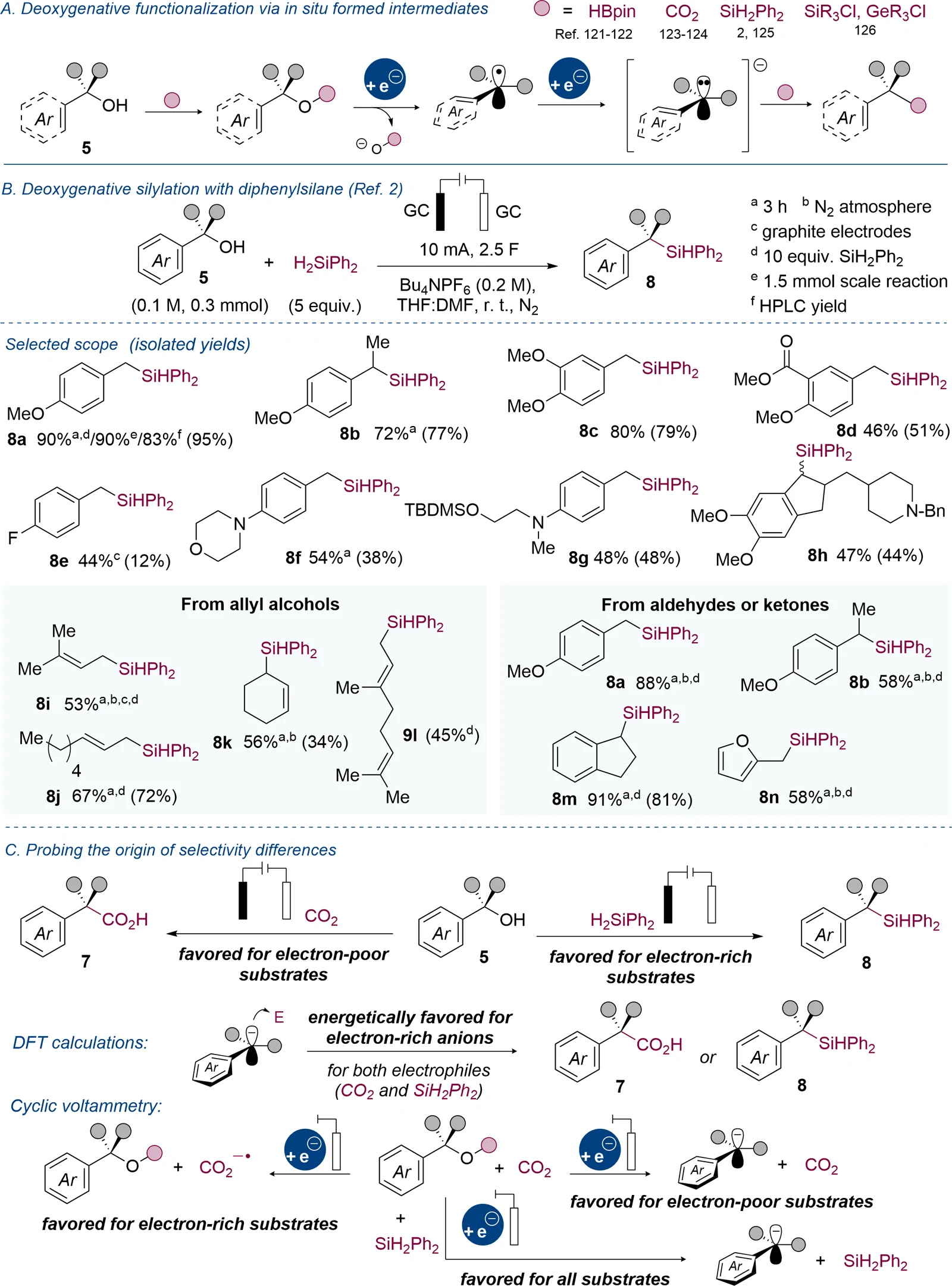
Deoxygenative C–C, C–B, and C–Si bond formations. A. In situ activation and radical–polar crossover pathways. B. Deoxygenative silylation of alcohols. C. Reactivity trends and reduction-potential matching. Adapted with permission from Ref. 2 licensed under CC BY 4.0. Copyright 2025 Wiley/the authors.

### 2.3 C–S Bond Cleavage

Carbon–sulfur bonds are widespread in molecules of natural and synthetic origin, including amino acids, pharmaceuticals, agrochemicals, fragrances and materials (Figure 7A).^127–132^ Their abundance, together with the polarized nature of the C–S bond, makes organosulfur compounds attractive precursors for reductive bond cleavage and subsequent functionalization.^133–136^ Desulfurative chemistry has traditionally relied on high-valent or charged sulfur derivatives, such as sulfones, sulfoxides and sulfonium salts, which can serve as radical or anionic precursors under thermal, transition-metal-catalyzed, photochemical or electrochemical conditions (Figure 7B).^137–146^ In contrast, low-valent sulfur compounds, including thioethers, thiols, disulfides and thioacetals, remain less explored as alkyl precursors in one-electron reductive manifolds, despite their broad availability and synthetic relevance.^147–149^ Electrochemical C–S bond cleavage can proceed through either oxidative or reductive pathways. In electrooxidative processes, initial formation of a radical cation can promote fragmentation into carbocationic or thiyl-radical-type intermediates, a strategy that has been used for the functionalization of sulfides, thioacetals, carbohydrates and peptides (Figure 7C).^148,150–154^ In contrast, electroreductive C–S bond cleavage typically relies on cathodic single-electron transfer to generate a radical anion, followed by fragmentation to give a carbon-centered radical and a thiolate (Figure 7D).^155–158^ Depending on the reduction potential of the radical intermediate and the reaction medium, these species can either undergo hydrogen atom/proton transfer, react with a radical acceptor, or accept a second electron to furnish a carbanion through a radical–polar crossover mechanism.^159–161,35^ The latter pathway is particularly attractive because it enables the generation of nucleophilic carbon intermediates from bench-stable organosulfur precursors without strong bases or organometallic reagents.

**Figure 7:**
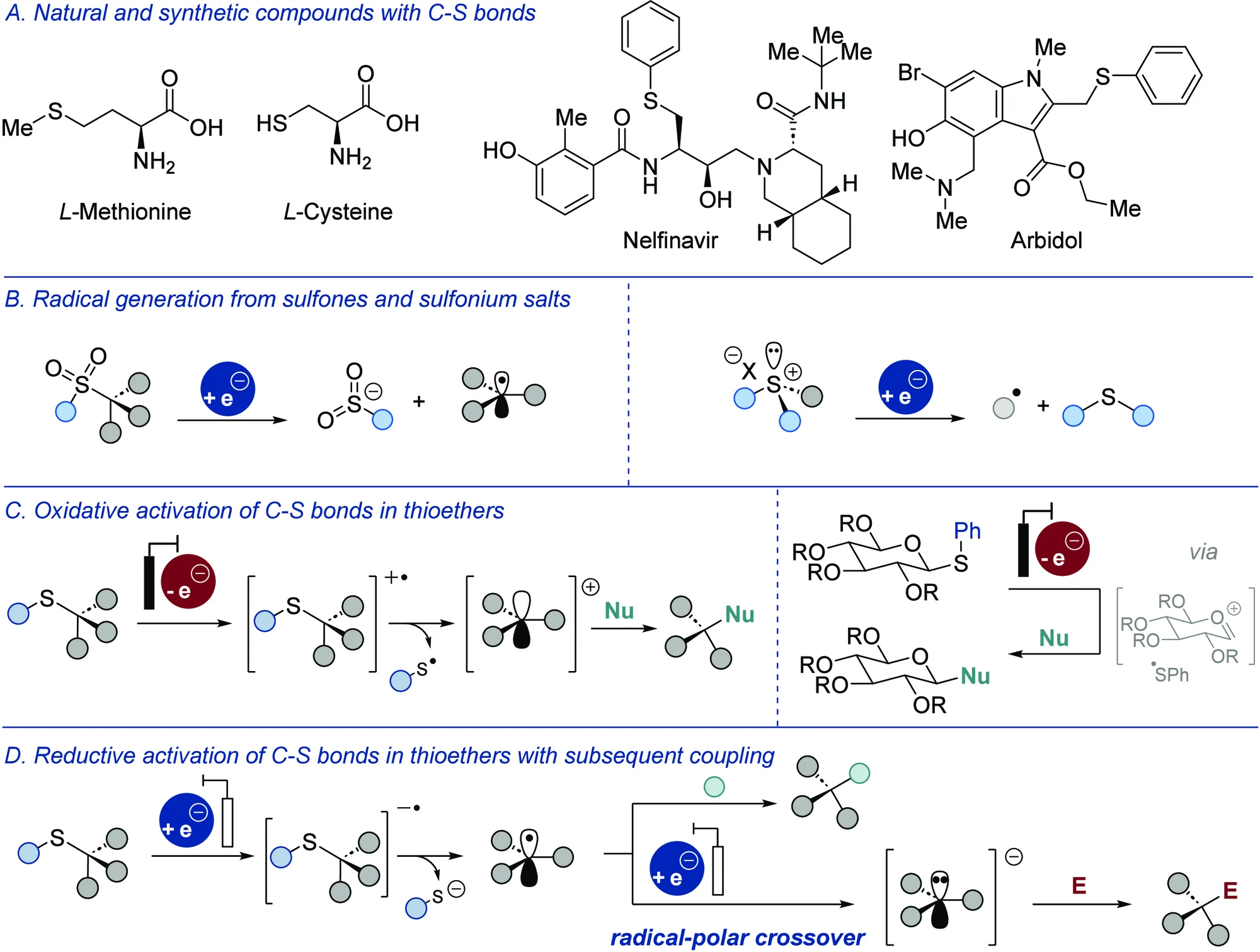
Sulfur-containing compounds and synthetic strategies for their cleavage. A. Representative organosulfur compounds. B. High-valent sulfur precursors. C. Electrooxidative C–S bond cleavage. D. Electroreductive C–S bond cleavage and radical–polar crossover.

Early examples of electroreductive C–S bond cleavage mainly focused on deprotection or hydrodesulfurization. Ives, Miyoshi, and co-workers reported electroreductive removal of S-benzyl groups from cysteine derivatives,^162,163^ while Young and co-workers described the related cleavage of benzylic picolyl groups from cysteine.^164^ Shono and co-workers developed an electrochemically driven elimination of β-hydroxy phenyl thioethers to form alkenes,^165^ and Torii and co-workers reported cleavage of 2-benzothiazolyl thioethers in π-activated positions.^166,167^ Pletcher and co-workers later demonstrated the reductive cleavage of benzyl thioethers to afford toluene derivatives in good yields.^168^ These studies established the feasibility of electrochemical C–S bond activation, but were mainly limited to reduction or elimination rather than trapping of the carbon intermediate. An early example pointing toward functionalization was reported by Guirado, Gallardo and co-workers, using 4-nitrobenzyl phenyl thioether as a model substrate for electrochemical CO_2_ activation and capture.^158^ Under N_2_ atmosphere, reduction led to C–S bond cleavage and hydrodesulfurization, whereas under CO_2_ the corresponding carboxylated product was obtained in low yield. This study highlighted a central challenge for reductive C–S functionalization: the carbon intermediate must be generated under conditions that are sufficiently reducing to cleave the C–S bond but not so reducing that the electrophile or product is consumed. Our group addressed this limitation by using the S-aryl group of aryl alkyl thioethers as a tunable electroauxiliary, shifting the reduction potential of the C–S bond cleavage step to enable chemoselective generation and trapping of alkyl radical/carbanion intermediates. The transformation was performed in a simple undivided cell under galvanostatic electrolysis, using a tin cathode and a sacrificial magnesium anode (Figure 8A).^3^ The reaction tolerated primary, secondary, tertiary, benzylic, and aliphatic thioethers, and could also be applied to substrates derived from cholestanol and cysteine. Replacement of MeCN with CD_3_CN enabled site-selective deuteration, consistent with formation of a reduced carbon intermediate that is protonated or deuterated by the reaction medium. Mechanistic studies supported initial electron transfer to the thioether, C­(sp^3^)–S bond scission to an alkyl radical and arylthiolate, and, when favored, further reduction of the radical to a carbanion (Figure 8D). The aryl group on sulfur was crucial for this reactivity, acting as an electroauxiliary that modulates the reduction potential of the thioether and enables milder, more chemoselective activation (Figure 8B). This effect was key for expanding the chemistry from simple hydrodesulfurization to productive C–C bond formation. Similar results of alkane products were obtained switching from the sacrificial Mg anode to the use of a carbon-based anode in the presence of TBABH_4_.^12^ Two complementary functionalization modes were demonstrated from thioethers. First, electrocarboxylation with CO_2_ provided arylacetic acids **7** through radical–polar crossover to a carbanion followed by trapping with CO_2_ (Figure 8C). Second, Giese-type addition to electron-deficient alkenes enabled desulfurative C­(sp^3^)–C­(sp^3^) bond formation (Figure 8D). In this case, careful matching of the reduction potentials of the thioether and alkene acceptor was necessary to avoid competitive reduction or hydrodimerization of the Michael acceptor.^169^ These studies indicate that thioethers are not simply less activated analogues of sulfoxides and sulfones but can provide complementary and synthetically useful reactivity in reductive desulfurative couplings.

**Figure 8:**
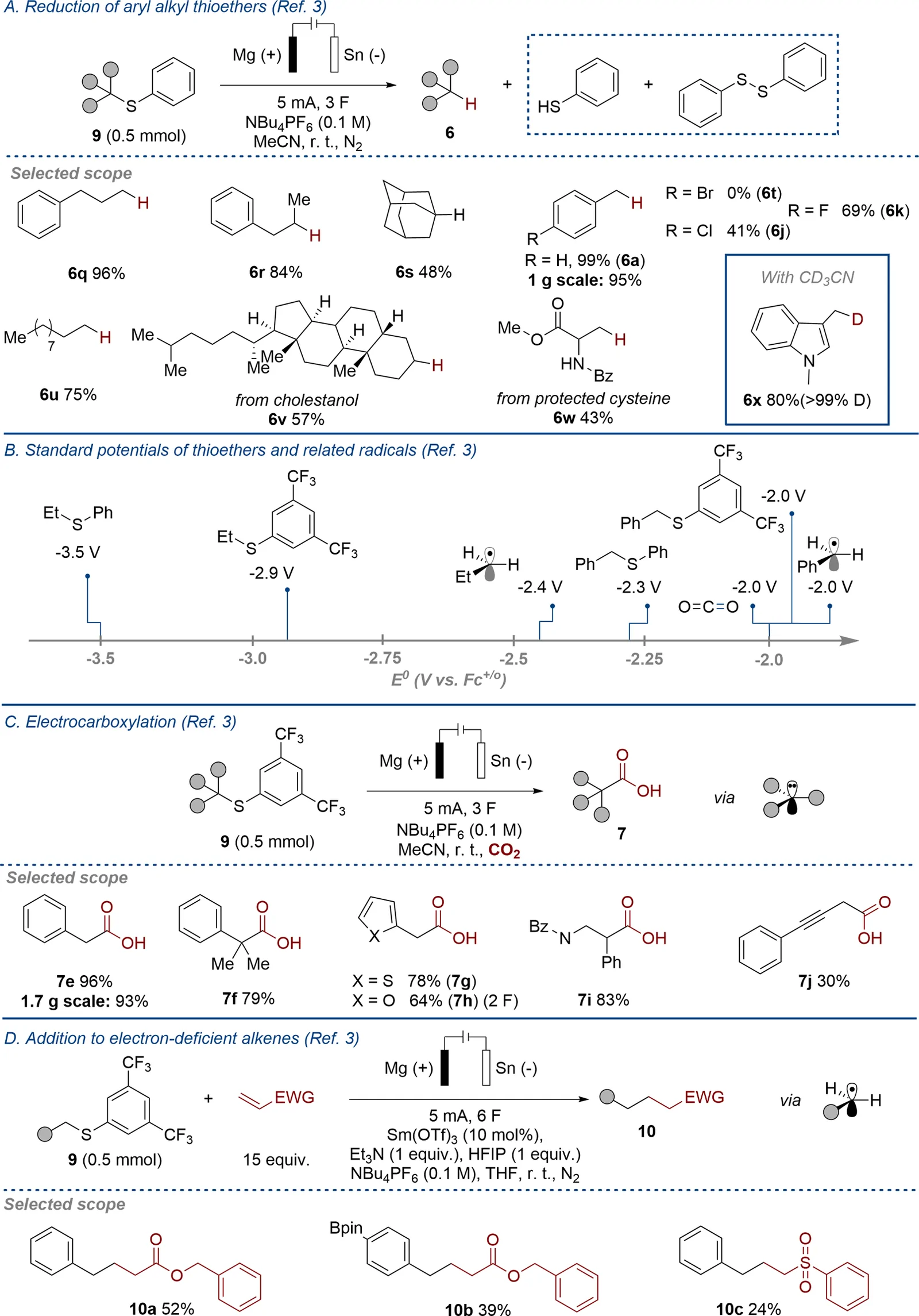
Reductive C–S bond cleavage in aryl alkyl thioethers. A. Hydrodesulfurization and deuteration. B. S-aryl electroauxiliary effect on potential. C. Electrocarboxylation with CO_2_. D. Giese-type C–C bond formation. Adapted with permission from Ref. 3, licensed under CC BY 4.0. Copyright 2023 Wiley/the authors.

The same strategy was later extended to thioacetals (**11**), valuable carbonyl-protecting and Corey–Seebach umpolung motifs whose classical cleavage often relies on Mozingo reduction or oxidative/radical protocols requiring harsh or toxic reagents.^170–174^ Earlier electrochemical work by Steckhan and co-workers showed that diphenyl dithioacetals could be reduced cathodically to thioethers in divided cells under constant potential conditions (Figure 9A).^175^ Building on the thioether platform, a more operationally simple galvanostatic protocol was developed for the metal-free electroreductive desulfurization of aryl thioacetals to alkanes in an undivided cell (Figure 9B).^176^ Mechanistic experiments supported the intermediacy of the corresponding thioether, consistent with a stepwise sequence in which two consecutive C–S bond-cleavage events convert thioacetals into alkanes (Figure 9C). The use of deuterated solvent further enabled site-selective deuterium incorporation, highlighting the broader potential of these reactions for isotopic labeling.

**Figure 9:**
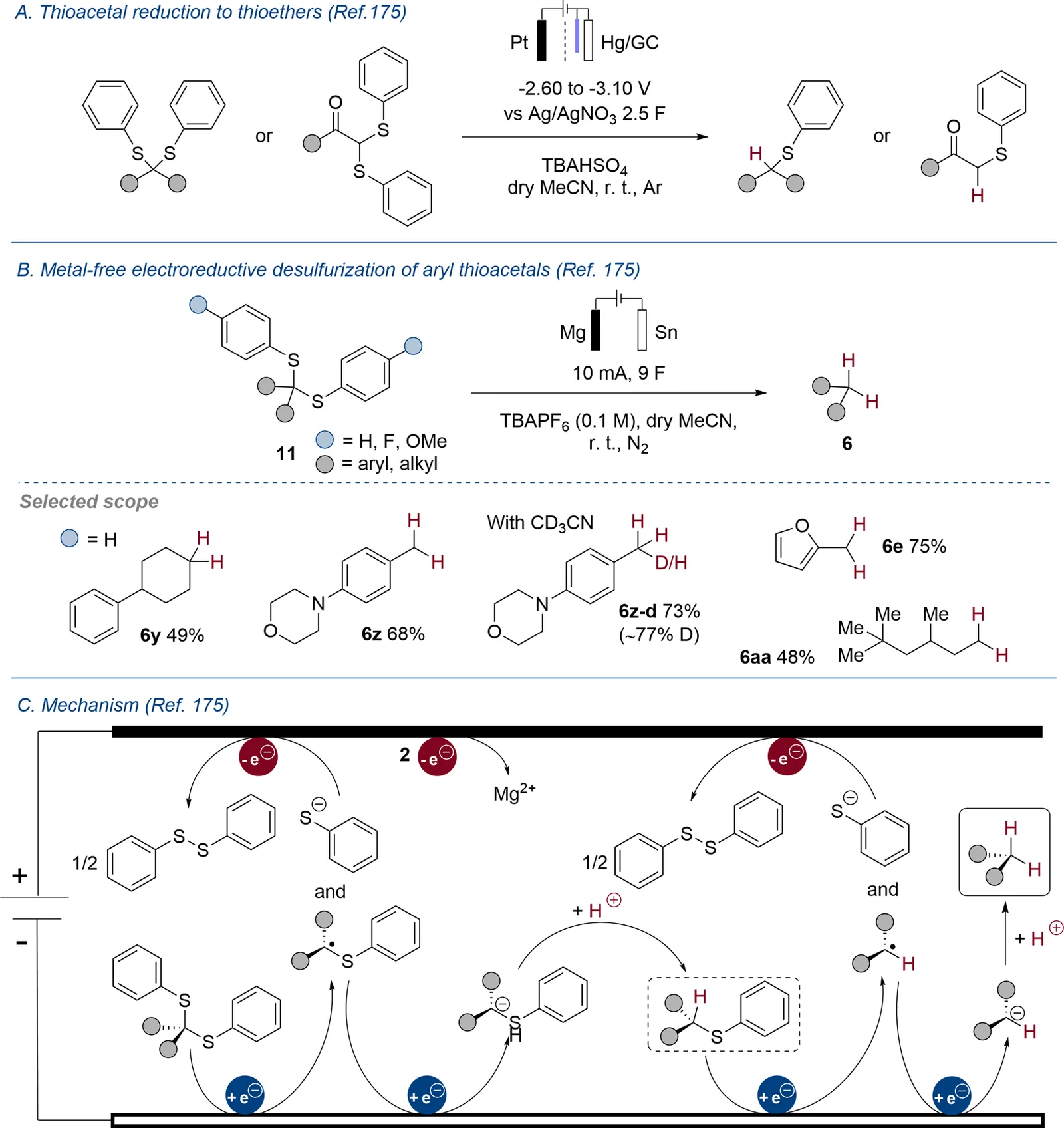
Electroreductive C–S bond cleavage in thioacetals. A. Precedent on cathodic thioacetal reduction. B. Electroreductive desulfurization of thioacetals. C. Mechanistic proposal for stepwise C–S bond cleavage. Adapted with permission from Ref. 176 licensed under CC BY 4.0. Copyright 2026 Wiley/the authors.

Finally, electroreductive C–S bond cleavage was expanded to C–B bond formation. Inspired by the cathodic stability and electrophilic behavior of pinacolborane, demonstrated by Lin and co-workers,^121^ our group developed a desulfurative borylation of thioethers, thioacetals, thiols and disulfides (Figure 10A).^4^ Using HBpin as the boron electrophile and tetrabutylammonium borohydride as both supporting electrolyte and sacrificial oxidizable additive,^12^ benzylic and allylic thiol derivatives were converted into synthetically valuable alkylboronic esters under mild electrochemical conditions. The protocol tolerated several reductively sensitive functional groups, heterocycles, natural-product-derived scaffolds, and pharmaceutically relevant fragments, and was scalable to gram quantities. Mechanistically, radical trapping experiments are consistent with C–S bond cleavage to a carbon-centered radical, followed by rapid radical–polar crossover to a carbanion and trapping by the reductively stable boron electrophile (Figure 10B). Together, these studies show that electroreductive C–S bond cleavage provides a versatile platform for converting abundant sulfur-containing compounds into radicals and carbanions under mild conditions. By controlling the electroauxiliary, electrode setup, counter reaction and electrophile compatibility, the strategy enables formation of C–H, C–D, C–C and C–B bonds from low-valent organosulfur precursors.

**Figure 10:**
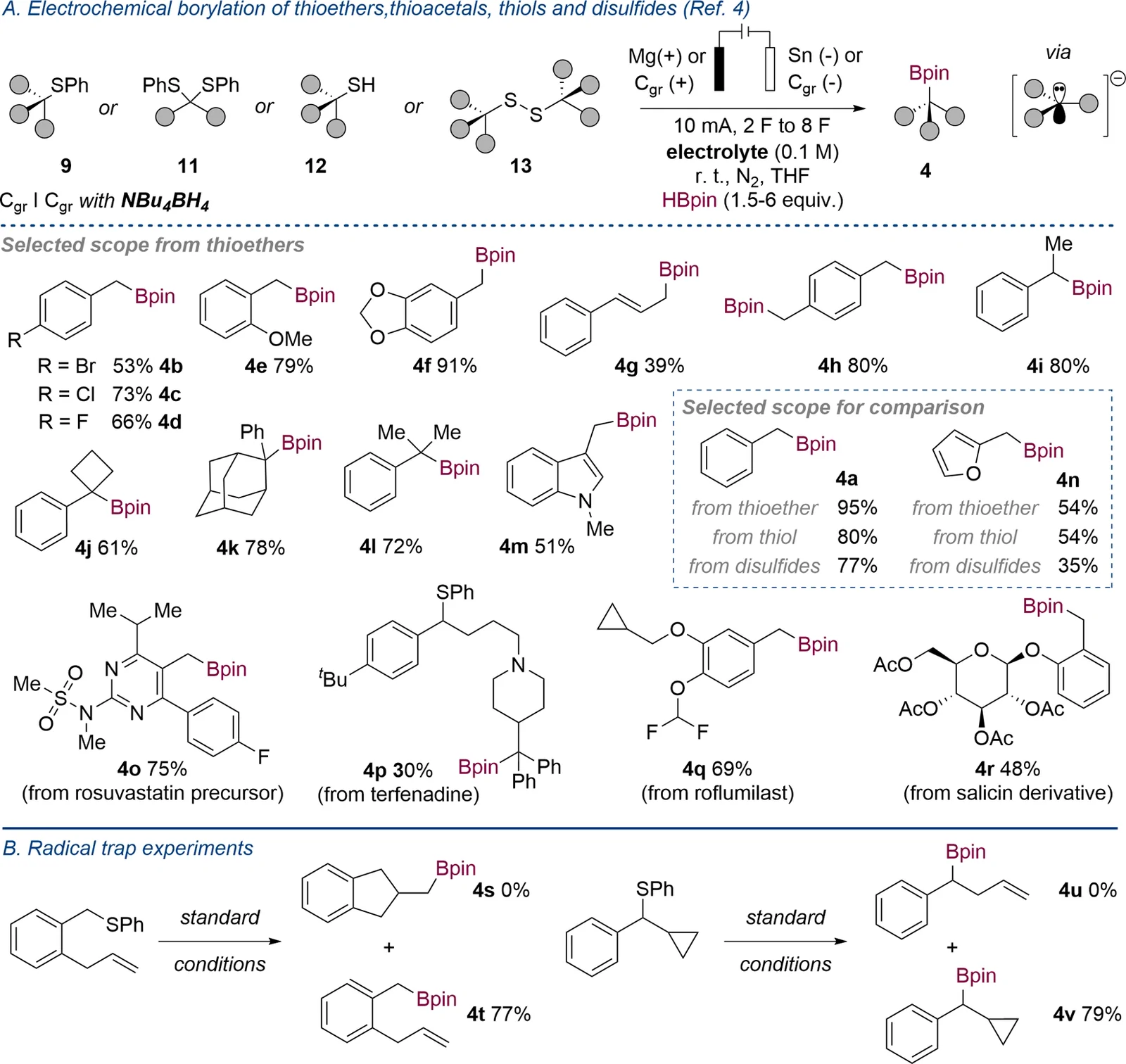
Desulfurative borylation of thiols and thiol derivatives. A. Electroreductive borylation of thioethers, thiols, and disulfides. B. Mechanistic studies supporting radical–polar crossover and carbanion trapping with HBpin. Adapted with permission from Ref. 4 licensed under CC BY 4.0. Copyright 2026 Springer Nature/the authors.

## 3 Conclusions and Outlook

The studies highlighted in this Account demonstrate how electroreduction can transform strong and abundant C–F, C–O and C–S bonds into programmable synthetic handles via umpolung in radical-polar crossover events. By combining cathodic bond cleavage with suitable reaction conditions and trapping partners, these stable bonds were converted into C–H, C–D, C–C, C–B and C–Si bonds in cross-couplings via intermediate formation of carbanionic intermediates. The cleavage of strong and relatively inert chemical bonds such as those highlighted in this Account is commonly challenging via traditional routes, typically requiring pyrophoric reagents at cryogenic temperatures. In this sense, the ability of electrochemistry to enable deeply reducing conditions to access carbanions at mild conditions and room temperature is highly useful.

Productive electroreductive cross-coupling normally depends on balancing substrate reduction and bond fragmentation with the cathodic stability of the acceptor, such that the latter is more reductively stable than the former. This scenario is challenging for many transformations operating at deep reduction potentials as the selection of electrophilic coupling partners stable under such conditions is limited. However, additional factors than reduction potential alone affect selectivity. For example, an unfavorable reduction of a substrate may be followed by fast and irreversible bond fragmentation that drives the transformation forward, effectively enabling the disfavored electron transfer to take place at a more anodic potential than expected.^177^ This principle has recently been suggested to be partially responsible for successful coupling of ketones and alkenes with mismatched redox potentials using photoredox catalysis,^178^ and may serve as a strategy to expand the selection of viable coupling partners in deep reduction electrolysis. Another underexplored opportunity lies in the interception of electrogenerated reactive intermediates with metal complexes to form organometallic reagents of Grignard or Negishi-type or catalytic alkyl metal complexes *in situ* for subsequent cross-couplings under milder conditions. Additional control may be achieved using modified cathodes with catalysts immobilized on their surfaces, creating local reactive environments that favor targeted reductions, bond cleavages and formations. Together, these emerging strategies may expand the viability of electroreductive generation and coupling of reactive species via C–F, C–O and C–S bond cleavage under mild conditions, broadening functional group tolerance and scope of coupling partners, removing the need for π-activated substrates and excess reagent concentrations and establishing the strategy as a general and practical platform for innovative molecular construction.
